# Rebiopsy Enhances Survival with Afatinib vs. Osimertinib in EGFR Exon 19 Deletion Non-Small Cell Lung Cancer: A Multicenter Study in Taiwan

**DOI:** 10.3390/curroncol32010036

**Published:** 2025-01-10

**Authors:** Jerry Shu-Hung Kuo, Cheng-Yu Chang, Shih-Chieh Chang, Yu-Feng Wei, Chung-Yu Chen

**Affiliations:** 1 Department of Internal Medicine, National Taiwan University Hospital Yunlin Branch, Yunlin County, Douliu City 640, Taiwan; 2 Division of Chest Medicine, Department of Internal Medicine, Far Eastern Memorial Hospital, New Taipei City 220, Taiwan; 3 Department of Nursing, Cardinal Tien Junior College of Healthcare and Management, New Taipei City 231, Taiwan; 4 Division of Chest Medicine, Department of Internal Medicine, National Yang-Ming Chiao Tung University Hospital, Yilan County, Yilan City 260, Taiwan; 11319@hosp.nycu.edu.tw; 5 Department of Internal Medicine, E-Da Cancer Hospital, I-Shou University, Kaohsiung City 824, Taiwan; 6 School of Medicine for International Students, College of Medicine, I-Shou University, Kaohsiung City 824, Taiwan; 7 Division of Pulmonary and Critical Care Medicine, Department of Internal Medicine, College of Medicine, National Taiwan University, Taipei City 100, Taiwan

**Keywords:** Afatinib, Osimertinib, EGFR mutation, lung cancer, rebiopsy

## Abstract

Background: Afatinib and Osimertinib are first-line treatments for EGFR-mutated advanced non-small cell lung cancer (NSCLC), but their comparative efficacies and the patient groups that benefit the most remain unclear. This multicenter retrospective study evaluated the efficacy of first-line Afatinib and Osimertinib in NSCLC patients with EGFR 19del and no brain metastases at diagnosis. Methods: The primary endpoints were time on treatment (ToT) and overall survival (OS). Survival analyses were performed for three groups: Afatinib followed by Osimertinib, Afatinib followed by other therapies, and Osimertinib (alone or followed by other therapies). Rebiopsy practices, including T790M mutation detection, were also analyzed in patients with disease progression on Afatinib. Results: Among 97 Afatinib-treated and 60 Osimertinib-treated patients, Osimertinib showed a significantly longer ToT (23.3 vs. 16.5 months; *p* = 0.007). Median OS was numerically higher for Afatinib with sequential Osimertinib (40.5 vs. 34.6 months for Osimertinib; *p* = 0.473). Osimertinib demonstrated advantages, with fewer brain metastases upon progression and fewer adverse effects. In the Afatinib group, 64% of patients with disease progression underwent rebiopsy, with 39% testing positive for T790M mutation and subsequently receiving Osimertinib. Rebiopsy was most frequently performed on the lung parenchyma using non-surgical methods. Conclusions: In this real-world study, Osimertinib achieved a significantly longer ToT compared to Afatinib in NSCLC patients with EGFR 19del and no brain metastases. The sequential use of Afatinib followed by Osimertinib showed a trend toward improved OS, highlighting the importance of rebiopsy for identifying T790M mutations to guide subsequent therapy.

## 1. Introduction

Targeted molecular treatments for advanced non-small cell lung cancer (NSCLC) have been revolutionized by the identification of oncogenic activation in specific tyrosine kinases, such as EGFR, ALK, and ROS1, using driver mutations as biomarkers [1,2]. This breakthrough has inspired further research into additional subsets of advanced NSCLC. Notably, in Asian populations, over 50% of advanced adenocarcinomas harbor activating epidermal growth factor receptor (EGFR) mutations. The two primary subtypes of EGFR mutations are the exon 19 deletion and the L858R point mutation in exon 21 [3].

EGFR tyrosine kinase inhibitors (TKIs) are the standard first-line therapy for EGFR-mutation-positive advanced NSCLC and are categorized into three generations: first-generation (Gefitinib and Erlotinib), second-generation (Afatinib and Dacomitinib), and third-generation (Osimertinib) [4]. Among these, Osimertinib has demonstrated superior intracranial activity compared to first- and second-generation TKIs, making it particularly effective in managing and preventing brain metastases [5]. Furthermore, Osimertinib is associated with fewer adverse effects [6] and is highly effective in overcoming acquired resistance caused by the T790M mutation, the most common molecular mechanism of resistance in patients experiencing cancer progression during treatment with first- or second-generation TKIs. This mutation accounts for approximately 50% to 60% of such cases [7,8,9,10,11].

First-line treatment with Afatinib, followed by second-line treatment with Osimertinib after tumor progression and detection of an acquired T790M mutation in a rebiopsy sample, results in a prolonged duration of treatment (Afatinib + Osimertinib: 24 to 27 months) and overall survival (median 36 months) [12,13]. For patients who do not undergo rebiopsy or lack a detectable T790M mutation, the guidelines recommend platinum-based chemotherapy as the next-line treatment. However, this approach is often challenging due to its associated side effects and is linked to shorter overall survival compared to patients with confirmed T790M mutations via rebiopsy, who benefit from sequential Osimertinib therapy in real-world settings [14].

In the phase IIB LUX-Lung 7 trial, Afatinib demonstrated superior progression-free survival (PFS) compared to first-generation Gefitinib in the first-line treatment of advanced NSCLC with EGFR mutations, along with a slight trend toward improved overall survival (OS) [15,16]. Similarly, the phase III FLAURA trial unequivocally showed that Osimertinib outperforms first-generation EGFR-TKIs such as Gefitinib and Erlotinib as a first-line treatment for EGFR-mutation-positive advanced NSCLC, with significant benefits in both PFS (hazard ratio [HR] for disease progression or death, 0.46; *p* < 0.001) and OS (HR for mortality, 0.80; *p* = 0.046) [6,17]. However, a recent multicenter cohort study (CJLSG1903) highlighted the potential benefits of a sequential treatment approach, where Afatinib followed by Osimertinib in patients with T790M mutations demonstrated prolonged overall survival compared to first-line Osimertinib in specific subpopulations, such as those with L858R mutations without brain metastases [18]. Currently, there is insufficient evidence to support a direct head-to-head comparison between Osimertinib and Afatinib as first-line therapies. The question of which treatment offers better clinical outcomes and the identification of patient subgroups that may derive the most benefit from each therapy remain inconclusive and warrant further investigation [13,19].

Since 2020, the Taiwan National Health Insurance (NHI) system has provided reimbursement for Osimertinib as a first-line therapy for stage IV lung adenocarcinoma with a positive EGFR mutation (Exon 19 deletion) and no initial central nervous system (CNS) metastasis. For other patients with untreated stage IV lung adenocarcinoma, the treatment options are limited to first- or second-generation TKIs, unless they opt to cover the cost of Osimertinib out-of-pocket, which amounts to approximately TWD 170,000 per month. Additionally, Osimertinib is reimbursed as a second-line therapy for EGFR-mutation-positive advanced NSCLC in cases of disease progression during Gefitinib, Erlotinib, or Afatinib treatment, provided that there is an acquired T790M mutation [20].

This research aims to comprehensively compare the clinical outcomes of first-line Afatinib and first-line Osimertinib in a real-world setting for patients meeting the Taiwan NHI reimbursement criteria for Osimertinib—advanced NSCLC with EGFR exon 19 deletion and no initial CNS metastasis. Additionally, the patient characteristics will be thoroughly described. Clinical data were analyzed to assess the rate of acquired T790M mutations in patients who experienced disease progression during first-line Afatinib therapy and subsequently underwent rebiopsy (tissue or liquid biopsy).

## 2. Materials and Methods

### 2.1. Study Design

This multicenter retrospective cohort study aimed to evaluate the efficacy of first-line Afatinib and first-line Osimertinib in stage IIIB-IV NSCLC patients harboring EGFR exon 19 deletions without CNS metastases at the time of initial diagnosis. Patients with other EGFR mutations, such as L858R or L791Q, and those with CNS metastases at diagnosis were excluded.

We retrospectively reviewed the medical records of adult patients who received first-line treatment with either Afatinib or Osimertinib and met the specified criteria across four medical centers in Taiwan. Patients in the Afatinib group were enrolled between July 2014 and May 2022, while those in the Osimertinib group were enrolled between April 2018 and June 2022. The data cut-off date was 30 September 2024.

This study was approved by the Research Ethics Committees of National Taiwan University Hospital Yunlin Branch, E-Da Hospital, Far Eastern Memorial Hospital, and National Yang-Ming University Hospital (approval number: 201611059RINB). Informed consent was waived due to the retrospective nature of this observational study, which relied on medical chart reviews for data analysis.

### 2.2. Patient Data Collection

The study collected comprehensive clinical data for each patient, including age, gender, Eastern Cooperative Oncology Group (ECOG) performance status, initial cancer stage, metastatic sites at diagnosis, comorbidities, smoking status, history of radiotherapy for local tumor control, and details regarding the types and duration of subsequent systemic therapies. All cases were reviewed, and the staging was re-evaluated to ensure consistency using the AJCC 8th edition TNM staging system. Treatment responses were classified as progressive disease (PD), stable disease (SD), partial response (PR), or complete remission (CR) according to the RECIST 1.1 criteria [21]. The objective response rate (ORR) was defined as the proportion of patients achieving the best response of CR or PR, while the disease control rate (DCR) included patients with CR, PR, or SD.

For patients who were treated with first-line Afatinib and experienced PD, rebiopsy was recommended, which could involve either tissue or liquid biopsy. If the rebiopsy specimen tested positive for an acquired T790M mutation during further EGFR mutation testing, Osimertinib was prescribed as the second-line treatment [4]. In cases where rebiopsy was not performed, or if the sample was T790M-negative or unsuitable for T790M testing, sequential systemic therapy, typically chemotherapy, was the most common treatment option.

This study meticulously recorded rebiopsy details, including the method, biopsy site, type of gene alteration examination, and T790M mutation status. For patients who did not undergo a rebiopsy, the main reasons for omission were also thoroughly documented.

The study primarily analyzed the time on treatment (ToT) and overall survival (OS) of patients receiving Afatinib or Osimertinib as first-line therapy for EGFR exon 19 deletion NSCLC. The primary endpoint was OS, while the ToT was defined as the duration from the initiation of first-line EGFR-TKI treatment to the discontinuation of the therapy. Additionally, survival analyses were conducted across three groups: Afatinib followed by Osimertinib, Afatinib followed by other systemic therapies, and Osimertinib as the sole treatment.

This study also compared the first-line treatments of Afatinib and Osimertinib, with a specific focus on adverse events. Adverse events were evaluated using the Common Terminology Criteria for Adverse Events (CTCAE) Version 5.0 [22]. The analysis assessed the occurrence and severity of adverse events during treatment with each drug and identified factors that could necessitate treatment discontinuation.

### 2.3. Statistical Analysis

Continuous variables were summarized as medians with ranges, while categorical variables were reported as frequencies and percentages. The Wilcoxon rank-sum (Mann–Whitney) test was used for comparing continuous variables between groups. Categorical variables, including differences in proportions or percentages, were compared using the Chi-square test or Fisher’s exact test, as appropriate. Cox proportional hazard regression models were employed to estimate hazard ratios for clinical characteristics and survival outcomes. Kaplan–Meier survival curves were generated for overall survival (OS) and time on treatment (ToT), with the Log-rank test being used to assess the statistical significance of differences in survival times between groups. Multivariate Cox regression models were applied to calculate the hazard ratios for OS and ToT while adjusting for potential confounding factors. A *p*-value of less than 0.05 was considered statistically significant. All data analyses were performed using STATA statistical software version 15 (StataCorp, College Station, TX, USA).

## 3. Results

### 3.1. Baseline Characteristics

A retrospective analysis was conducted on 157 patients with stage IIIB-IV NSCLC harboring EGFR exon 19 deletions without brain metastases. These patients were treated across four medical centers in Taiwan. In the first-line setting, 97 patients received Afatinib between July 2014 and May 2022, while 60 patients were administered Osimertinib between April 2018 and March 2022. Table 1 presents the baseline characteristics of the patients included in this study.

The median age of the Afatinib group was 63 years (interquartile range [IQR], 55–71), significantly younger than the Osimertinib group, where the median age was 69 years (IQR, 62–77.5; *p* = 0.013). Additionally, a significantly higher proportion of patients in the Afatinib group had an ECOG performance status (PS) score of 0–1 (86%) compared to the Osimertinib group (72%; *p* = 0.004). No notable differences were observed between the two groups regarding comorbidities, sites of metastasis at diagnosis, cancer stage, or likelihood of undergoing local radiotherapy.

### 3.2. Comparative Analysis of Survival Benefits: Afatinib vs. Osimertinib

The median follow-up time was 33.2 months (IQR, 19.7–46.8) in the Afatinib group and 32.2 months (IQR, 18.7–38.7) in the Osimertinib group. The objective response rate (ORR) was 61.0% in the Afatinib group and 75.4% in the Osimertinib group. The disease control rate (DCR) was 94.7% in the Afatinib group and 98.3% in the Osimertinib group.

**Figure 1:**
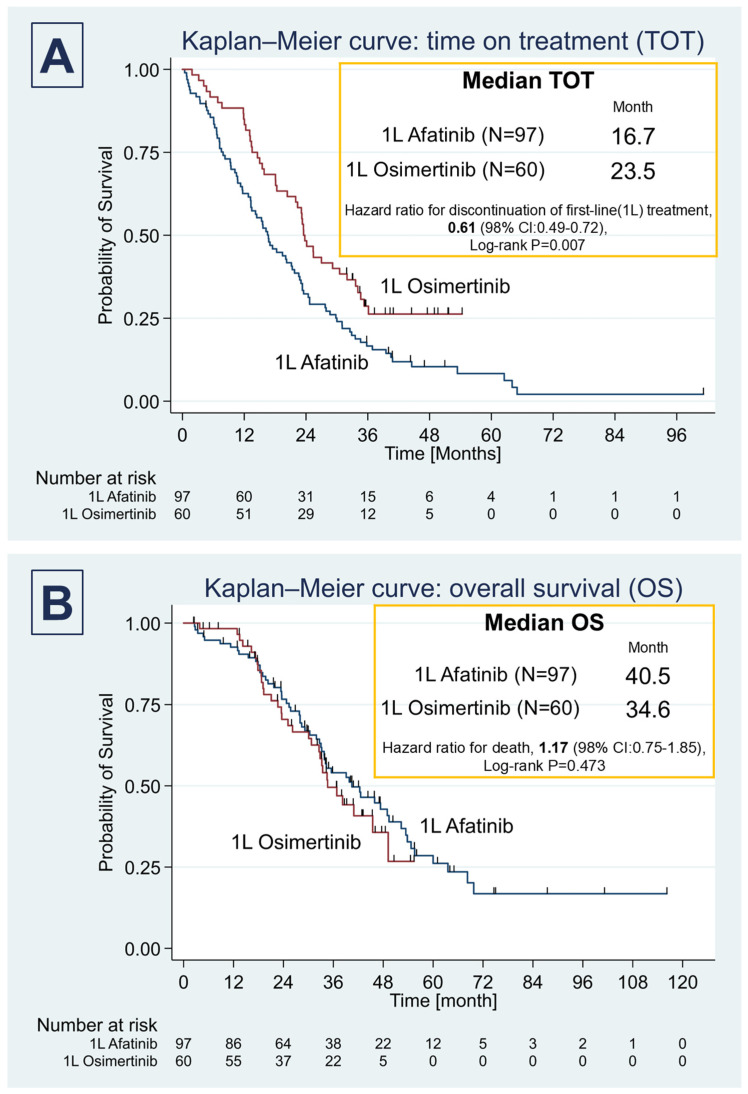
Kaplan–Meier survival curves for time on treatment (TOT) and overall survival (OS)**.** (**A**) The median time on treatment (TOT) in the Afatinib cohort was 16.7 months (interquartile range [IQR], 7.9–30.0), significantly shorter than in the Osimertinib cohort, where it was 23.8 months (IQR,13.4–not reached) (hazard ratio for discontinuation of first-line [1 L] treatment, 0.61 (98% CI: 0.49–0.72); Log-rank *p* = 0.007). (**B**) However, while the OS in the Afatinib group appeared higher at 40.5 months (IQR, 25.4–63.6) compared to the 34.6 months (IQR, 22.7–not reached) in the Osimertinib group, this difference was not statistically significant (hazard ratio for death, 1.17 (98% CI: 0.75–1.85); Log-rank *p* = 0.473).

The median time on treatment (ToT) in the Afatinib group was 16.7 months (IQR, 7.9–30.0), significantly shorter than the 23.5 months (IQR, 13.5–not reached) observed in the Osimertinib group (*p* = 0.007). However, the overall survival (OS) was not significantly different between the groups. The median OS in the Afatinib group was 40.5 months (IQR, 25.4–63.6) compared to 34.6 months (IQR, 22.7–not reached) in the Osimertinib group (Log-rank *p* = 0.473) (Figure 1A,B).

In the multivariate analysis, Osimertinib significantly reduced the risk of disease progression compared to Afatinib (HR: 0.59, 95% CI: 0.37–0.96, *p* = 0.033). Patients with poor ECOG PS (PS ≥ 2) had a higher risk of death compared to patients with better ECOG PS (PS: 0–1) (HR: 1.96, 95% CI: 1.14–3.39, *p* = 0.015) (Supplementary Tables S1 and S2).

### 3.3. Subgroup Analysis of First-Line Afatinib

**Figure 2:**
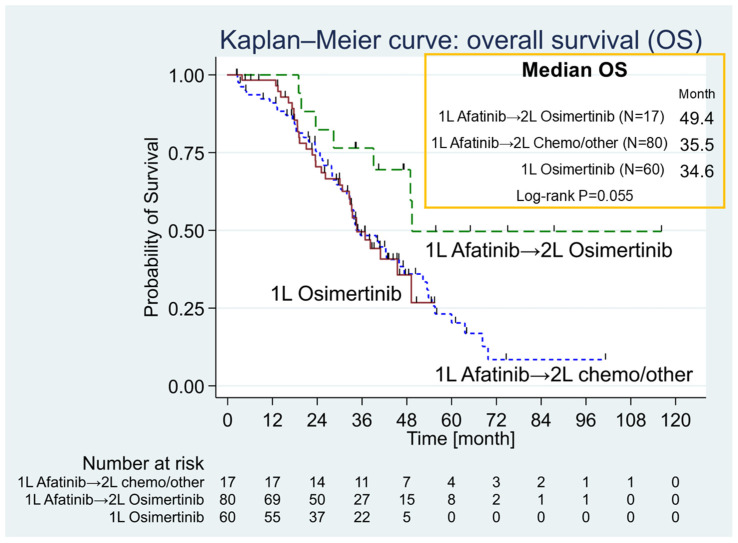
Comparative Kaplan–Meier analysis of overall survival (OS) across three distinct treatment groups. The Kaplan–Meier curve analysis was conducted to evaluate overall survival (OS) across three distinct groups. Upon subdividing the Afatinib group into two categories, it was noted that the subgroup receiving first-line [1 L] Afatinib followed by second-line [2 L] Osimertinib exhibited a promising trend, with a median OS of 49.4 months (green dashed line), surpassing both the 1 L Afatinib followed by 2 L chemotherapy or other treatment subgroup (blue short-dashed line; median OS: 35.5 months) and the first-line Osimertinib subgroup (red line; median OS: 34.6 months). The differences among the three groups were not statistically significant (Log-rank *p* = 0.055).

The Afatinib group was further divided into two subgroups: patients who received first-line Afatinib followed by Osimertinib, and those who received first-line Afatinib followed by chemotherapy or other treatments. A trend toward longer OS was observed in the subgroup treated with first-line Afatinib followed by Osimertinib (median OS: 49.4 months; IQR: 39.1–not reached) compared to first-line Afatinib followed by chemotherapy or other treatments (median OS: 35.5 months; IQR: 24.7–55.6) and first-line Osimertinib (median OS: 34.6 months; IQR: 22.7–not reached). However, the difference among the three groups did not reach statistical significance (Log-rank *p* = 0.055) (Figure 2).

The analysis of progression sites following first-line therapy revealed distinct patterns in the two groups. Afatinib treatment was associated with a higher incidence of brain metastases upon disease progression (13.1%, 8 of 61 patients) compared to Osimertinib (6.7%, 2 of 30 patients; Fisher’s exact test *p* = 0.488). Other progression sites included bone, pleura, lung, liver, and adrenal glands, with no statistically significant differences between the groups (Supplementary Table S3).

### 3.4. Exploring Clinical Decision-Making and Rebiopsy Trends Following Disease Progression on Afatinib Therapy

**Figure 3:**
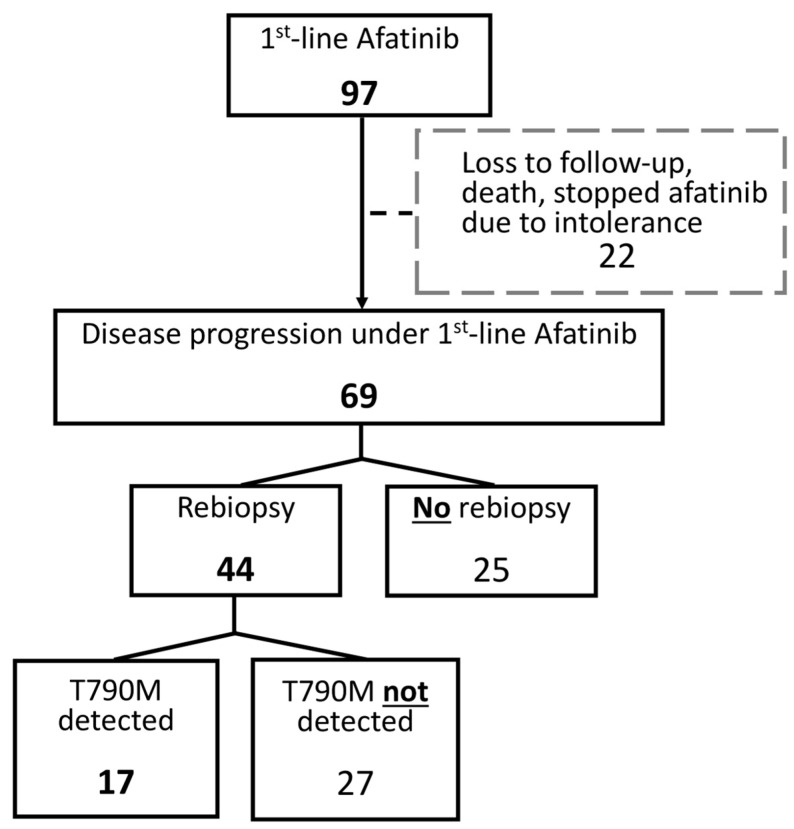
Flow diagram of patients with/without rebiopsy in first-line Afatinib group. In the Afatinib cohort, 69 out of 97 patients experienced disease progression during first-line treatment after excluding those who were lost to follow-up, deceased, or discontinued their use due to intolerable side effects. Among these, 64% (44 patients) underwent rebiopsy, revealing 17 cases with confirmed acquisition of the T790M mutation. Subsequently, all identified patients received Osimertinib.

Of the 97 patients in the Afatinib group, 69 who experienced disease progression during first-line Afatinib treatment were included in this analysis. Patients who were lost to follow-up, deceased, or discontinued Afatinib due to intolerable side effects were excluded. Among these 69 patients, 44 (64%) underwent rebiopsy, and 17 (39%) of those rebiopsies revealed an acquired T790M mutation. All 17 patients who were confirmed to have the T790M mutation were subsequently treated with Osimertinib (Figure 3).

The timing of disease progression was categorized into three periods based on key milestones in Osimertinib’s regulatory approval and reimbursement status: 1. Before 31 December 2016—approved by the US FDA but not yet certified in Taiwan. 2. 1 January 2017–31 March 2020—certified in Taiwan but not covered by National Health Insurance. 3. After 1 April 2020—conditionally covered by Taiwan’s National Health Insurance.

The proportion of patients undergoing rebiopsy increased significantly over these time periods, rising from 38% to 55% and then to 90%, reflecting enhanced accessibility and awareness of rebiopsy practices. The Roche Cobas^®^ EGFR Test v2 was the preferred method for detecting gene alterations.

**Figure 4:**
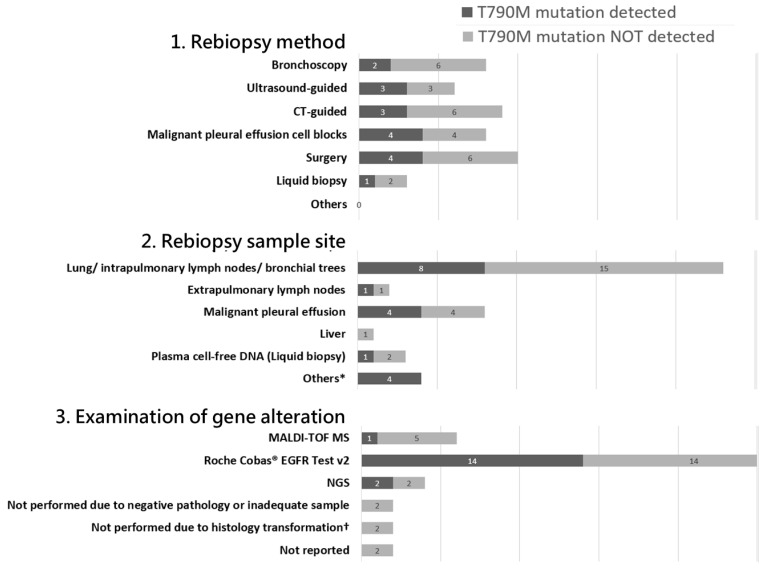
Biopsy methods/sample sites/types of gene alteration examination among the 44 patients receiving rebiopsy for detection of acquired T790M mutation. Upon a detailed analysis of cases undergoing rebiopsy, non-surgical methods were found to predominate. These included bronchoscopic biopsy, ultrasound-guided biopsy, CT-guided biopsy, and pleural effusion drainage. The most frequently targeted sites for rebiopsy were the lung parenchyma, intrapulmonary lymph nodes, and bronchial trees. MALDI-TOF MS, Matrix-Assisted Laser Desorption Ionization–Time-of-Flight Mass Spectrometry; NGS, next-generation sequencing. * Metastatic sites: bladder tumor, right neck mass, right subphrenic tumor, pleural tumor; † both are small cell transformations.

A detailed analysis of rebiopsy cases revealed that most patients underwent non-surgical methods, including bronchoscopic biopsy, ultrasound-guided biopsy, CT-guided biopsy, and pleural effusion drainage. The lung parenchyma and intrapulmonary lymph nodes were the most common biopsy sites (Figure 4). The primary reason for not performing rebiopsy was identified as “Not feasible for rebiopsy”, encompassing factors such as poor functional status, multiple comorbidities, or technical challenges in accessing the biopsy site (Table 2).

### 3.5. Differential Incidence Rates and Profiles of Adverse Effects (AEs) in Afatinib Versus Osimertinib Treatment Cohorts

Adverse effects (AEs) were reported in 91.2% of patients in the Afatinib cohort, with Grade 3 or higher events occurring in 39.4% of cases. In comparison, AEs were reported in 80.0% of patients in the Osimertinib cohort, with Grade 3 or higher events observed in 23.3% of cases. The most common AE in both groups was diarrhea, affecting 66% of patients in the Afatinib group versus 35% in the Osimertinib group. Other prevalent AEs in the Afatinib group included rash and paronychia, while fatigue was more frequently observed in the Osimertinib group. A higher proportion of patients in the Afatinib group required dose reductions (45%) compared to the Osimertinib group (20%). However, there were no statistically significant differences in the frequency of treatment holds or in the rates of treatment discontinuation due to AEs between the two groups.

## 4. Discussion

This study compared the efficacy of first-line Afatinib and Osimertinib in patients with advanced NSCLC harboring EGFR exon 19 deletions without baseline CNS metastases, focusing on real-world outcomes under Taiwan’s NHI reimbursement regulations. While Osimertinib demonstrated a significantly longer time on treatment (ToT), the overall survival (OS) in the Afatinib group, particularly in patients transitioning to second-line Osimertinib, exhibited a promising trend toward being numerically longer. These findings underscore the need for personalized treatment strategies and emphasize the critical role of rebiopsy in optimizing outcomes for patients who are treated with Afatinib [1].

Several retrospective studies have shown no significant differences in OS between first-line Afatinib and Osimertinib. Notably, Osimertinib demonstrated a potential OS advantage in patients with baseline brain metastases, as highlighted by a Taiwanese single-center study [23] and a nationwide registry in the Netherlands [24]. Similarly, a small-scale study found no significant differences in OS or PFS between the two treatments [25]. The Giotag study demonstrated that sequential therapy with Afatinib followed by Osimertinib achieved a median OS of 37.6 months in the overall cohort and 44.8 months in the Asian subgroup [13]. In our study, sequential Afatinib and Osimertinib yielded a numerically higher median OS of 49.4 months. However, this result may be influenced by “immortal time bias”, as events for patients transitioning to Osimertinib after Afatinib could only be recorded post-rebiopsy and treatment initiation, potentially skewing the results in favor of sequential therapy.

In this study, patients in the Osimertinib group had a higher median age and a lower proportion with ECOG PS 0–1 compared to the Afatinib group, reflecting real-world physician preferences to prescribe Afatinib to younger, healthier patients to preserve the option of detecting T790M mutations through rebiopsy upon progression [23]. Older patients with EGFR-mutated NSCLC and good performance status, along with fewer than three metastatic sites, have been associated with longer progression-free survival (PFS) and overall survival (OS) when treated with EGFR-TKIs [26]. Additionally, Afatinib has been linked to longer PFS, with younger age and an absence of brain metastases correlating with better OS outcomes [13]. These findings suggest that the sequential use of second-line Osimertinib may extend chemotherapy-free survival. However, the proportion of patients with disease progression on first-line Afatinib who underwent rebiopsy was relatively low in our study, with only 44 of 69 patients (64%) completing the procedure. This rate, along with the 41–72% reported in other Taiwan studies [27,28,29], is slightly lower than the 71–77% observed in Korea [30,31]. Such discrepancies highlight regional and systemic variations in rebiopsy practices, underscoring the need to address barriers and standardize approaches to optimize outcomes across different healthcare settings.

In the Afatinib group, the most common reasons for not performing a rebiopsy were “not feasible for rebiopsy”, often due to poor functional status, comorbidities, or challenges in accessing biopsy sites, and “patient or family refusal.” The enrollment period for the Afatinib group (July 2014 to May 2022) spanned significant advancements in NSCLC-targeted therapies and biopsy techniques, including the introduction of liquid biopsy and next-generation sequencing (NGS). These advancements have improved the effectiveness and safety of rebiopsy, increasing its acceptance for detecting actionable mutations like T790M after disease progression on first-line TKIs. Taiwan’s National Health Insurance (NHI) policy changes in April 2020, enabling reimbursement for Osimertinib as a second-line therapy in T790M-positive cases, further contributed to the significant rise in rebiopsy rates, particularly after 2020. The rebiopsy rate in our cohort aligns with other studies in Taiwan, reflecting a growing feasibility and acceptance in real-world practice [27,28,29]. The AURA3 trial underscored the critical role of rebiopsy by demonstrating that second-line Osimertinib significantly improves efficacy, prolongs response duration, and reduces adverse events compared to chemotherapy for patients with T790M mutations [11].

Advancements in medical technology, expanded reimbursement criteria, and growing evidence supporting Osimertinib’s efficacy have encouraged the adoption of invasive procedures (e.g., bronchoscopy, surgery) and advanced testing (e.g., NGS) to detect actionable mutations like T790M. In real-world practice, the prescribing rates of Osimertinib vary significantly across regions, most likely due to differences in cost and reimbursement policies [32]. For example, China’s inclusion of Osimertinib in reimbursement programs increased its use [33], while only 3.7% of EGFR-positive metastatic NSCLC patients in South Korea receive it as first-line therapy due to limited coverage [34]. However, further research is needed to determine the specific impact of Taiwan’s NHI reimbursement policy changes on treatment practices and clinical outcomes [35,36]. In April 2022, Taiwan NHI limited first-line Osimertinib coverage to patients with CNS metastases due to economic considerations. This policy change may reduce the accessibility of Osimertinib for broader first-line use and warrants close monitoring to evaluate its impact on patient outcomes. For L858R-mutated NSCLC, first-line Osimertinib has not demonstrated significant advantages over first- or second-generation EGFR TKIs. Studies, such as those by Ito et al. [18], show comparable efficacy between Afatinib and Osimertinib for L858R patients without brain metastases. However, out-of-pocket costs for Osimertinib in Taiwan may limit its usage in this subgroup, reducing the availability of real-world data and introducing bias. Addressing these disparities is essential to ensure equitable access and optimize treatment outcomes.

Our study found a higher, though not statistically significant, rate of brain metastasis progression in patients who were treated with first-line Afatinib compared to Osimertinib. In the FLAURA trial, Osimertinib’s superior CNS penetration reduced the progression risk (HR: 0.46) and achieved a higher intracranial response rate (66% vs. 43%) [6,17,37], supported by real-world data [38]. Afatinib, however, has shown comparable PFS to Erlotinib and superior PFS to Gefitinib in Del19 mutant NSCLC with baseline brain metastases, and similar rates of systemic and CNS-only progression compared to Gefitinib/Erlotinib in patients without brain metastases [39,40,41]. A retrospective study reported no significant differences in median PFS, ORR, or CNS progression between Afatinib and Osimertinib in patients without baseline brain metastasis [23]. Additionally, the incidence of new metastatic lesions was comparable between the groups (13.1% for Afatinib vs. 6.7% for Osimertinib) in our study, although another study demonstrated all CNS metastases during therapy occurred in the Osimertinib group [25].

This study’s retrospective design introduces potential biases, including a variability in treatment selection influenced by physician preferences and patient characteristics. The Osimertinib group had a higher median age and lower ECOG PS than the Afatinib group, reflecting a tendency to prescribe Afatinib to younger, healthier patients. The lack of randomization limits direct comparisons between groups, while the long enrollment period for the Afatinib cohort (2014–2022) spanned significant advancements in diagnostic and therapeutic practices, potentially affecting outcomes over time. Immortal time bias, where patients require rebiopsy and progression documentation before transitioning to second-line Osimertinib, may have influenced the observed OS trends. The limited use of liquid biopsy further restricted mutation detection in patients who were unfit for invasive procedures. Additionally, focusing on exon 19 deletion mutations limits the generalizability of these findings to other EGFR subtypes, including L858R and other uncommon mutations [42]. Considering the biological differences in T790M mutation acquisition rates, including L858R patients in future studies could provide a more comprehensive understanding. Since October 2024, Taiwan’s National Health Insurance (NHI) has started reimbursing Osimertinib as a first-line treatment for EGFR exon 21 L858R mutations. Further studies stratified by EGFR mutation subtype are warranted to validate these findings. Changes in Taiwan NHI’s reimbursement policies, including the 2022 revision restricting first-line Osimertinib coverage to patients with CNS metastases, highlight the need for ongoing monitoring of real-world treatment patterns. Policymakers should ensure equitable access to advanced diagnostics and therapies to maximize survival benefits across diverse patient populations.

## 5. Conclusions

In summary, this study underscores the efficacy of both Afatinib and Osimertinib as first-line therapies for EGFR-mutant NSCLC, with Osimertinib offering a longer ToT and Afatinib showing potential for extended OS with sequential second-line therapy. Rebiopsy remains a cornerstone in managing disease progression, enabling the identification of actionable mutations like T790M and facilitating transitions to more effective therapies. Advancements in diagnostic technologies, combined with supportive NHI reimbursement policies, have the potential to further enhance patient outcomes by bridging gaps in access to personalized cancer care. Further prospective studies are essential to validate these findings and optimize treatment strategies, although obtaining funding for such research remains a considerable challenge.

### Clinical Practice Findings

- This study underscores the clinical utility of both Afatinib and Osimertinib as first-line therapies for EGFR-mutant NSCLC patients without CNS metastases. Osimertinib demonstrated a significantly longer time on treatment (ToT) compared to Afatinib, while Afatinib followed by Osimertinib showed a promising trend toward longer overall survival (OS).
- Rebiopsy plays a pivotal role in identifying actionable mutations, such as T790M, in patients with disease progression on Afatinib. This facilitates the transition to second-line Osimertinib, which can significantly extend survival outcomes.
- Diagnostic advancements: Incorporating advanced diagnostic tools, such as liquid biopsy and next-generation sequencing (NGS), could improve mutation detection rates, especially for patients who are unfit for invasive procedures.

## Figures and Tables

**Table 1:** Baseline characteristics of the study cohort (*n* = 157).

	Afatinib Group	Osimertinib Group	*p*-Value
Patient, *n*	97	60	
Age, year, median (IQR)	63 (55–71)	69 (62–77.5)	0.013
<50, *n* (%)	13 (13)	5 (8)	
50–64, *n* (%)	37 (38)	15(25)	
≥65, *n* (%)	47 (48)	40(67)	
Sex, female, *n* (%)	42 (43)	32 (53)	0.251
ECOG PS 0–1, *n* (%)	83 (86)	43 (72)	0.004
Never smoked, *n* (%)	64 (66)	38 (63)	0.734
At least one comorbidity, *n* (%)	70 (72)	44 (73)	1
Hypertension, *n* (%)	43 (44)	27 (45)	1
Diabetes mellitus, *n* (%)	26 (27)	14 (23)	0.708
Chronic kidney disease, *n* (%)	4 (4.1)	5 (8.3)	0.304
Chronic liver diseases, *n* (%)	10 (10)	7 (12)	0.797
Chronic pulmonary obstructive disease, *n* (%)	7 (7)	6 (10)	0.562
Initial cancer stage, *n* (%)			0.252
Stage IIIB or IIIC	6 (6)	1 (2)	
Stage IV	91 (94)	59 (98)	
Metastatic sites at the time of initial diagnosis, *n* (%)			
Lung-to-lung	35 (36)	22 (37)	1
Liver	9 (9)	5 (8)	0.785
Bone	31 (32)	23 (38)	0.49
Malignant pleural effusion	33 (34)	21 (35)	1
Undergoing any local radiotherapy, *n* (%)	22 (23)	9 (15)	0.304

ECOG PS, Eastern Cooperative Oncology Group Performance Status; IQR, interquartile range.

**Table 2:** The main reason for not performing rebiopsy among patients who experienced disease progression under first-line Afatinib (*N* = 29).

	*N* (%)
1. Death or limited life span	4 (16%)
2. Not feasible for rebiopsy (poor functional status or multiple comorbidities or difficult approach)	10 (40%)
3. Patient or family’s refusal	5 (20%)
4. Choice of second-line systemic therapy other than TKIs due to cancer rapid progression	2 (8%)
5. Not reported	8 (32%)

TKIs, tyrosine kinase inhibitors.

## Data Availability

The original contributions presented in this study are included in the article/Supplementary Material. Further inquiries can be directed to the corresponding author.

## Supplementary Materials

The following supporting information can be downloaded at https://www.mdpi.com/article/10.3390/curroncol32010036/s1: Supplementary Table S1. Multivariate analyses of clinical factors affecting time on treatment. Supplementary Table S2. Multivariate analyses of clinical factors affecting overall survival. Supplementary Table S3. Disease progression after first-line treatment with Afatinib and Osimertinib.
