# Supplementary material for: Rebiopsy Enhances Survival with Afatinib vs. Osimertinib in EGFR Exon 19 Deletion Non-Small Cell Lung Cancer: A Multicenter Study in Taiwan
Source: Curr Oncol. 2025 Jan 10;32(1):36. doi: 10.3390/curroncol32010036 (PMC11763488; doi:10.3390/curroncol32010036)
Supplement: Supplementary file 1 [file curroncol-32-00036-s001.zip › curroncol-3378228-supplementary.pdf]

Supplementary Table S1. Multivariate analyses of clinical factors affect time-on-treatment.

	**HR (95% CI)**	***p* value**
First line EGFR TKI		
Afatinib	Reference	
Osimertinib	0.58 (0.39 - 0.86)	.006
Age		
<65 year	Reference	
>= 65 year	1.25 (0.86 – 1.82)	.243
Gender		
Female	Reference	
Male	1.29 (0.82 – 2.02)	.275
ECOG		
PS 0-1	Reference	
PS >=2	1.52 (0.97 – 2.36)	.065
Smoking status		
Never-smoker	Reference	
Former/current smoker	0.76 (0.49 –1.18)	.221
Initial stage (Stage III B/C vs. IV)	0.55 (0.23 – 1.31)	.181
Metastasis at initial diagnosis		
Lung to lung	1.09 (0.74 – 1.62)	.652
Bone	1.15 (0.77 – 1.70)	.495
Liver	1.56 (0.89 – 2.74)	.122
Malignant pleural effusion	1.16 (0.79 – 1.70)	.456
Local RT		
No	Reference	
Yes	0.79 (0.50 – 1.23)	.301

TKI, tyrosine kinase inhibitors; ECOG PS, Eastern Cooperative Oncology Group Performance Status; RT, radiotherapy

Supplementary Table S2. Multivariate analyses of clinical factors affect overall survival.

	**HR (95% CI)**	***p* value**
First line EGFR TKI		
Afatinib	Reference	
Osimertinib	1.16 (0.72 –1.87)	.544
Age		
< 65 year	Reference	
>= 65 year	1.29 (0.81 – 2.05)	.277
Gender		
Female	Reference	
Male	1.72 (0.97 – 3.05)	.063
ECOG		
PS 0-1	Reference	
PS >=2	1.90 (1.09 – 3.30)	.022
Smoking status		
Never-smoker	Reference	

Former/current smoker	0.65 (0.38 – 1.12)	.120
Initial stage (Stage III B/C vs. IV)	1.15 (0.39 – 3.37)	.797
Metastasis at initial diagnosis		
Lung to lung	1.11 (0.68 – 1.80)	.682
Bone	1.17 (0.71 – 1.94)	.532
Liver	0.52 (0.20 – 1.34)	.175
Malignant pleural effusion	1.82 (1.13 – 2.95)	**.014**
Local RT		
No	Reference	
Yes	0.95 (0.55 – 1.62)	.847

TKI, tyrosine kinase inhibitors; ECOG PS, Eastern Cooperative Oncology Group Performance Status; RT, radiotherapy

Supplementary Table S3. Disease Progression after First-line treatment of Afatinib and Osimertinib.

**PD site**	**Afatinib n = 61 (%)**	**Osimertinib n = 30 (%)**
Brain	8 (13.1)	2 (6.7)
Bone	1 (1.6)	3 (10.0)
Pleura	20 (32.7)	7 (23.3)
Lung / Intrathoracic lymph node	34(55.7)	22 (73.3)
Liver	5 (8.2)	2 (6.7)
Adrenal	2 (3.3)	0 (0.0)

PD: progression disease
